# Are circulating CEA immune complexes a prognostic marker in patients with carcinoma of the gastrointestinal tract?

**DOI:** 10.1038/bjc.1980.199

**Published:** 1980-07

**Authors:** H. J. Staab, F. A. Anderer, E. Stumpf, R. Fischer

## Abstract

CEA immune complexes and free CEA were determined to 363 patients with histologically confirmed adenocarcinoma of the gastrointestinal tract before surgery and in a post-operative follow-up. Circulating CEA immune complexes (CEA-IC) could be detected preoperatively in 89 patients. Incidence of CEA-IC increased with increasing tumour extension; 72/89 patients with CEA-IC showed already metastatic disease progression, 40/89 had nonresectable tumours. Patients with preoperative CEA-IC had a poorer prognosis than patients without CEA-IC but with high levels of free CEA, or CEA-negative patients. The appearance of CEA-IC with consecutive increases in the postoperative follow-up indicated disease recurrence. In 32/55 relapse cases, circulating CEA-IC were detected postoperatively, all 32 cases developing metastatic spread of disease.


*Br. J. Cancer* (1980) **42**, 26

### ARE CIRCULATING CEA IMMUNE COMPLEXES A PROGNOSTIC MARKER IN PATIENTS WITH CARCINOMA OF THE GASTROINTESTINAL TRACT?

H. J. STAAB*, F. A. ANDERER*, E. STUMPF† AND R. FISCHER†

*From the *Friedrich-Miescher-Laboratorium der Max-Planck-Gesellschaft* 7400 *Tübingen and the †Chirurgische Klinik,* 7000 *Stuttgart-Bad Cannstatt, Theodor Veielstrasse* 90, *West Germany*

Received 28 January 1980 Accepted 24 March 1980

**Summary.—CEA immune complexes and free CEA were determined in 363 patients with histologically confirmed adenocarcinoma of the gastrointestinal tract before surgery and in a post-operative follow-up. Circulating CEA immune complexes (CEA-IC) could be detected preoperatively in 89 patients. Incidence of CEA-IC increased with increasing tumour extension; 72/89 patients with CEA-IC showed already metastatic disease progression, 40/89 had nonresectable tumours. Patients with preoperative CEA-IC had a poorer prognosis than patients without CEA-IC but with high levels of free CEA, or CEA-negative patients. The appearance of CEA-IC with consecutive increases in the postoperative follow-up indicated disease recurrence. In 32/55 relapse cases, circulating CEA-IC were detected postoperatively, all 32 cases developing metastatic spread of disease.**

ANTIBODIES reacting with carcinoembryonic antigen (CEA) have originally been demonstrated in patients with nonmetastatic digestive-system cancers and in pregnant women by Gold (1967) using an indirect haemagglutination technique. After the development of highly sensitive radioimmunoassay methods the existence of circulating anti-CEA antibodies could be confirmed (Gold *et al.*, 1972; McSween, 1975). Evidence for the presence of CEA immune complexes (CEA-IC) in glomerular deposits has been given for a case of colonic carcinoma with nephrotic syndrome (Costanza *et al.*, 1973) as well as for a case of pancreatic carcinoma where a complex containing immunoglobulin M and a CEA cross-reacting component could be isolated from the ascitic fluid (Harvey *et al.*, 1978). Recently, it was also shown that the sera of patients with gastrointestinal (GI) cancer contained a fraction of CEA-IC in which CEA was bound to IgM and partly also to IgG, and which could be separated from the free CEA. The binding of CEA to IgM and IgG was demonstrated by displacement experiments with a radioactive CEA marker using radioimmunodiffusion. Free CEA-binding immunoglobulins could not be detected (Kapsopoulou-Dominos & Anderer, 1979*a*).

In the last decade there was controversy about the autoantigenicity of CEA. Various groups using different techniques were unable to confirm the existence of circulating anti-CEA antibodies (Collatz *et al.*, 1971; LoGerfo *et al.*, 1972; Sorokin *et al.*, 1973). One group, however, could demonstrate circulating antibodies against NCA (nonspecific cross-reacting antigen), a glycoprotein present in normal human tissue and strongly cross-reacting with CEA by means of a "common site" antibody (Von Kleist *et al.*, 1972, 1978). The authors stressed the point that anti-

Address reprint requests to F. A. Anderer, Friedrch-Miescher-Laboratorium der Max-Planck-Gesellschaft, 7400 Tübingen, Spemannstrasse 37–39, West-Germany.

CEA antibodies might be specific antibodies against NCA.

Antibodies, specific for CEA or NCA, in the presence of antigen excess should be found predominantly in the fraction of immune complexes. The detection of CEA immune complexes in sera of patients with gastrointestinal cancer (Kapsopoulou-Dominos & Anderer, 1979*a*) provokes the question whether these complexes may block tumour-cell destruction by immune lymphocytes, thus enhancing tumour growth. Evidence that "blocking antibodies" are antigen-antibody complexes has been given by several groups (Sjögren *et al.*, 1971; Baldwin *et al.*, 1972; Jose & Seshadri, 1974).

In our present study, performed in the years 1976 to 1979, we examined the possible correlation of preoperative CEA-IC with the degree of tumour extension, the postoperative changes of the amount of CEA bound to immunoglobulins during a 3-year follow-up study and the survival rate of patients with CEA-IC. We used a routine determination of CEA bound to immunoglobulins (Kapsopoulou-Dominos & Anderer 1979*b*) which is based on the fact that immune complexes are precipitated together with the bulk of other serum proteins when perchloric acid is added to the serum. CEA present in the perchloric acid precipitates was found to be bound to IgM and IgG, which could be demonstrated in radioimmunological displacement experiments. The amount of CEA corresponded fairly well to that obtained by column fractionation (Kapsopoulou-Dominos & Anderer, 1979*a,b*).

### MATERIALS AND METHODS

*Patients and sera.*—363 patients (m/f = 1·5) were registered for a follow-up of the serum concentrations of free CEA and CEA bound in immune complexes. All patients were treated by surgery and had histologically proven adenocarcinomata of the rectum (80), colon (120), stomach (144) or pancreas (19). For the characterization of the extent of the tumours, we used the TNM classification of the International Union Against Cancer (1978).

Sera were obtained from blood samples taken a few days before and 8-10 days after operation, and thereafter at intervals of 2-3 months.

*Radioimmunoassay.*—The concentration of free CEA in the sera was determined after perchloric acid extraction by the Hansen Z-gel method (Hansen *et al.*, 1971) using the CEA-Roche RIA test kit (Roche Diagnostics, Basel, Switzerland). Variations in the anti-CEA serum batches of the CEA Roche RIA test kit were controlled on the basis of our own internal CEA standards.

The CEA-IC were found exclusively in the perchloric-acid precipitate (Kapsopoulou-Dominos & Anderer, 1979*b*) provided that dilution of the original serum (0·5 ml + 2 ml saline) addition of perchloric acid and sedimentation of the resulting precipitate (2500 *g*, 20 min) was carried out without delay. After removal of the perchloric-acid extracts the amount of CEA bound in immune complexes was determined in the perchloric-acid sediment as follows: The wall of the test tube containing the sediment obtained from 0·5 ml of original serum, was gently rinsed with 5 ml $H_2O$ without perturbing the sediment. The supernatant fluid was decanted and the sediment dissolved in 2ml 2M Tris solution yielding about pH 9·5. Thereafter, an aliquot of 0·5 ml was brought to 5 ml by adding saline and dialysed against 0·01M ammonium acetate (pH 6·8) followed by determination of CEA with the Roche RIA test without further perchloric-acid treatment. The sensitivity of the approach for the determination of CEA-IC was mainly limited by the criteria of the CEA Roche-RIA test. The lower limit for reproducible determinations of CEA in the perchloric-acid sediment was found to be 3·5 ng/ml original serum.

Optimal separation of free CEA and CEA-IC by perchloric-acid precipitation was dependent on the overall protein concentration of the serum. Sera with a high level of free CEA were prediluted with an adequate control serum before precipitation of the CEA-IC with perchloric acid, since predilution with buffer alone yielded a decreased amount of perchloric-acid precipitate, and also a decreased amount of CEA-IC in the precipitates, possibly due to formation of acid-soluble complexes (Kapsopoulou-Dominos & Anderer, 1979*b*). Control sera contained no

CEA-IC and were generally obtained from healthy persons.

Perchloric-acid precipitation of the CEA-IC together with the bulk of serum proteins includes the risk of unspecific co-precipitation and inclusion of free CEA in the precipitate. In a separate study this possibility was investigated, using undiluted sera which contained various concentrations of exogenous CEA and radioiodinated CEA (1 ng) as a marker. The amount of free CEA in the perchloric-acid precipitates varied between 3 and 10% at all CEA concentrations between 30 and 1000 ng CEA/ml serum. These findings gave the basis for a correction of the amount of CEA bound in immune complexes present in perchloric acid precipitates.

In our control sera the intra-assay variance ($1\sigma$) for the determination of free CEA was $4{\cdot}8 \pm 0{\cdot}4$ ng/ml serum and of complex bound CEA $4{\cdot}5 \pm 1{\cdot}3$ ng/ml serum.

### RESULTS

### *Incidence of preoperative CEA immune complexes*

Circulating CEA-IC were detected in 89 of 363 patients with histologically proven adenocarcinoma of the GI tract. All of these 89 patients also had a high serum concentration of free CEA, except 13 with a free CEA level less than 2 ng/ml serum. In Fig. 1 the preoperative values of free CEA (abscissa) and the values of CEA-IC (ordinate) are given in a double logarithmic plot for each patient. Patients (111/363) with neither free CEA levels $> 2$ ng/ml nor CEA bound in immune complexes $> 3{\cdot}5$ ng CEA/ml were not listed. Among the $CEA^+$ patients we observed cases with very high concentrations of free CEA and no CEA-IC as well as cases with low values of free CEA and a high amount of CEA-IC.

To answer the question whether the presence of circulating CEA-IC before surgery has any clinical relevance to the prognosis of the patients we tried to correlate a set of clinical parameters with the following 3 categories of preoperative CEA concentrations: (1) free CEA $< 2$ ng/ml and no detectable CEA-IC; (2) free CEA high but no detectable CEA-IC; (3) CEA-IC present. As clinical parameters we used location of the primary tumour,

FIG. 1.—Double logarithmic plot of the preoperative concentration of free CEA *vs.* CEA bound in immune complexes (CEA-IC) for individual patients with histologically proven adenocarcinoma of the gastrointestinal (GI) tract. Values of CEA-IC are corrected for unspecific coprecipitation of 10% of the free CEA.

TABLE.—*Correlation of the clinical status of patients with adenocarcinoma of the GI tract with preoperative levels of free CEA and CEA–IC*

| | Patients with ng free CEA/ng CEA–IC/ml serum | | | | |
|---|---|---|---|---|---|
| | < 2/< 3·5 | ≥ 2/< 3·5 | any ≥ 3·5 | Total | % with CEA–IC |
| Location of the primary carcinoma | | | | | |
| Rectum | 19 | 42 | 19 | 80 | 24 |
| Colon | 11 | 23 | 9 | 43 | 21 |
| Sigmoid colon | 26 | 27 | 24 | 77 | 31 |
| Stomach | 53 | 63 | 28 | 144 | 19 |
| Pancreas | 2 | 8 | 9 | 19 | 47 |
| Total | 111 | 163 | 89 | 363 | |
| Tumour extent (TNM) | | | | | |
| $T \neq 0$, N,M = 0 | 54 | 62 | 17 | 133 | 13 |
| T,$N \neq 0$; M = 0 | 45 | 65 | 31 | 141 | 22 |
| M = 1 | 9 | 34 | 41 | 84 | 49 |
| Total | 108 | 161 | 89 | 358 | |
| Surgical treatment | | | | | |
| Palliative | 36 | 62 | 32 | 130 | 27 |
| Nonresectable | 6 | 29 | 40 | 75 | 53 |
| Age distribution | | | | | |
| < 60 | 28 | 40 | 22 | 90 | 24 |
| 60–70 | 43 | 55 | 33 | 131 | 25 |
| > 70 | 40 | 68 | 34 | 142 | 24 |
| Total | 111 | 163 | 89 | 363 | |

tumour extent according to the TNM classification, surgical treatment (palliative or nonresectable) and age distribution. The corresponding data are systematically listed in the Table. The most interesting result was that the portion of patients with circulating CEA-IC increased with increasing tumour extent. In about half the cases with widespread tumour growth (M = 1) and nonresectable tumours, CEA-IC could be detected preoperatively. This portion was 4 × that in the cases with local tumours without lymphnode metastasis. In the group of 17 patients with resectable tumours and circulating CEA-IC, 2 cases staged T 1, 4 cases T 2, 5 cases T 3 and 6 cases T 4 were recorded. 68/75 cases with nonresectable tumours were staged T 4, mostly with metastases. The location of the primary tumours appeared to have no significant influence on the incidence of CEA-IC, except for carcinoma of the pancreas. Furthermore, the presence of preoperative CEA-IC was independent of age.

*Survival of patients with preoperative CEA-IC*

All of our 363 patients now have a follow-up of free CEA and CEA-IC for at least 2 years, and some for more than 3 years. A comparison of the survival of patients after surgery yields more detailed information on the prognosis of these patients. We selected 3 subgroups specified by the following preoperative CEA criteria: (1) patients with CEA-IC; (2) patients with high levels of free CEA but with no detectable circulating CEA-IC; (3) $CEA^-$ patients. The survival curves in Fig. 2A show that patients with stomach cancer and preoperative CEA-IC have a poor prognosis, with a half life of about 120 days after surgery, compared with patients with only high levels of free CEA (half life 300 days) and patients with neither high free CEA nor CEA-IC (600 days). The survival curves of patients with colorectal cancer (Fig. 2B) indicate a distinctly better prognosis. Even when CEA-IC could be detected preoperatively, this group of

FIG. 2.—Survival curves of patients with histologically proven adenocarcinoma of the GI tract and with the following preoperative CEA criteria: ○—○ patients with neither high levels of free CEA nor CEA–IC; ●—● patients with no CEA–IC but high levels of free CEA; ▲—▲ patients with CEA immune complexes. A: Patients with carcinoma of the stomach; B. patients with colorectal carcinoma.

patients showed a half life of about 420 days, and the group with only high levels of free CEA, about 720 days.

The survival curves of patients with only pathological concentrations of free CEA were significantly different from those of CEA$^-$ patients in the range of 300 to 1200 days in cases with stomach cancer, and in the entire range of postoperative surveillance in cases with colorectal cancer. Significant differences in survival between patients with CEA-IC and with patients with only pathologically high levels of free CEA were obtained up to 720 days in cases with stomach cancer, and in the range between 180 and 840 days in cases with colorectal cancer.

*Postoperative follow-up of free CEA and CEA-IC*

In this part of the study we investigated the appearance of circulating CEA-IC as a marker for early detection of disease recurrence. Only patients who underwent curative resection as judged from the situs and the results of the histological examination were subject of the follow-up. Therefore this group excludes most of the patients with preoperatively detectable CEA-IC, who predominantly had non-resectable tumours (40/89) or received only palliative treatment (32/89).

In the group of 158 patients with curative resections we have had up to date 57 cases of disease recurrence with consecutively increasing levels of free CEA. CEA-IC were detected in the sera of 34/57 patients up to 9 months before clinical diagnosis was possible (4/17 patients with primary resection of stomach cancer, 29/39 with primary colorectal cancer, 1/1 with pancreatic cancer). All 9 patients with local recurrence had no detectable circulating CEA-IC (5 patients with stomach cancer, 4 with colorectal cancer). Patients with detectable CEA-IC always had distant metastatic spread. In Fig. 3 the time course of free CEA and CEA-IC

FIG. 3.—CEA surveillance diagram of a 73-year-old female with an extirpated adenocarcinoma of the rectum who developed a localized recurrence 14 months after surgery. After successful second-look surgery the patient remained free of disease for further 16 months, when a peritoneal carcinosis was diagnosed, accompanied by increases in free CEA (broken line) and CEA-IC (solid line).

FIG. 4.—CEA surveillance diagram of an 82-year-old male with resected stomach cancer who developed a peritoneal carcinosis with a rapid increase of CEA-IC (solid line) followed by a sudden decrease to zero before death (free CEA: broken line).

is given for a case with a resected primary carcinoma of the rectum and second-look surgery for a local recurrence. The patient had no CEA-IC until 30 months after the second-look operation, when the patient developed a peritoneal carcinosis. It is noteworthy that the time course of CEA-IC in cases with recurrent disease did not always parallel the course of free CEA. In 7/32 cases of disease recurrence and detectable CEA-IC the amount of complex-bound CEA was distinctly higher than the amount of free CEA, 2–6 months before clinical diagnosis, partly immediately after primary resection (2 stomach cancer, 5 colorectal cancer).

We also found intermediate increases of CEA-IC up to 2 months after surgery, not necessarily linked to a simultaneous increase of free CEA, which were usually found in cases with lymphnode metastasis. Sometimes, similar intermediate increases later in the follow-up could also be detected, for instance, in 2 cases with resected primary carcinomas of the stomach and rectum and a temporary infection of the urological tract.

In the course of our study, 100 patients with far-advanced tumour growth could be followed up to death by serial determinations of CEA-IC and free CEA. In this group 51% of the patients had steadily increasing amounts of CEA-IC several months before death; 39% of these patients died during that phase of increase and 12% showed a dramatic decrease shortly before death, without an analogous change in the concentration of free CEA. In Fig. 4 the follow-up diagram is given for a patient who had only palliative treatment of a primary carcinoma of the stomach and developed a peritoneal carcinosis. The CEA-IC started to increase 4 months after surgery and dropped to zero after 18 months, possibly due to the beginning of immunological anergy. The patient died 25 months after operation without a significant change of the level of free CEA.

The time courses of free CEA in this group of patients showed in 76% of cases a steady, in some cases dramatic increase of free CEA in the final phase, and in the remainder no significant changes in the concentration of free CEA before death.

### DISCUSSION

The data obtained in our study indicate that the preoperative detection of circulating CEA-IC can be used as a prognostic marker in patients with adenocarcinoma of the GI tract. At the time of primary resection sera of 89/363 patients contained detectable amounts of CEA-IC and 72 of these 89 patients already showed metastatic disease progression, as judged from the situs and the histological examinations. The postoperative survival curves of the patients with preoperative CEA-IC agree with a poorer prognosis than that of patients who had only high levels of free CEA. In addition, the appearance of circulating CEA-IC in the postoperative follow-up also indicates a poor prognosis, *i.e.* all patients with CEA complexes developed metastatic disease. In some cases the increase of CEA-IC occurred before the increase of free CEA.

The mechanism by which circulating CEA-IC are influencing disease progression can be interpreted in terms of "immune lymphocyte blocking" and enhancement of tumour growth, as had been reported for other systems by several groups (Sjögren *et al.*, 1971; Baldwin *et al.*, 1972; Jose & Seshadri, 1974). Preliminary studies by us with a limited number of patients indicate that in about 20% of the cases CEA-specific lymphocyte cytotoxicity can be blocked with CEA-IC (work in progress). This mechanism need not be unique, since metastatic disease progression was also seen without detectable amounts of CEA-IC. On the other hand, one has to bear in mind that our method of detecting CEA-IC is restricted to the serum, and is possibly not sensitive enough to detect trace amounts which might be of immunological relevance.

The mechanism of induction of anti-CEA antibodies cannot be substantiated on the basis of our present knowledge. Autoantigenicity of CEA is suggested by the fact that the presence of circulating CEA-IC is predominantly associated with metastatic disease progression, which could be understood in terms of enhancement of tumour growth by "immune lymphocyte blocking", and by analogy with a number of reports which have already demonstrated that animals bearing very different tumours have mounted an immune response not only against tumour-specific antigens but also against embryonic components (Baldwin *et al.*, 1974; Baldwin & Embleton, 1974; Steele *et al.*, 1975; Zöller *et al.*, 1976; Tagliabue *et al.*, 1979). An alternative explanation resides in the possibility that the formation of CEA-IC is a result of cross-reacting antibodies. However, autoantibodies against NCA (Collatz *et al.*, 1971; Von Kleist & Burtin, 1966) seem not to be involved, since increases in the concentration of NCA are of less clinical value in indicating tumour progression than those of CEA (Von Kleist *et al.*, 1977). This would most likely also apply to NCA immune complexes. On the other hand NCA-specific antibodies should preferably bind to NCA which is present in a manyfold excess in the serum as compared to CEA (Von Kleist *et al.*, 1977) and according to the expected affinity of these antibodies all free NCA should be bound in complexes before CEA complexes are formed.

The authors thank Mrs S. Glock for excellent technical assistance and Mrs E. Wehrle for the management of data processing.

### REFERENCES

Baldwin, R. W., Price, M. R. & Robins, R. A. (1972) Blocking of lymphocyte-mediated cytotoxicity for rat hepatoma cells by tumor-specific antigen-antibody complexes. *Nature, New Biol.*, **238,** 185.

Baldwin, R. W., Glaves, D. & Vose, B. M. (1974) Immunogenicity of embryonic antigens associated with chemically induced rat tumors. *Int. J. Cancer*, **13,** 135.

Baldwin, R. W. & Embleton, M. J. (1974) Neoantigens on spontaneous and carcinogen-induced rat tumors defined by *in vitro* lymphocytotoxicity assays. *Int. J. Cancer*, **13,** 433.

Collatz, E., Von Kleist, S. & Burtin, P. (1971) Further investigations of circulating antibodies in colon cancer patients on the autoantigenicity of the carcinoembryonic antigen. *Int. J. Cancer*, **8,** 298.

Costanza, M. E., Pinn, V., Schwartz, R. S. & Nathanson, L. (1973) Carcinoembryonic antigen-antibody complexes in a patient with colonic carcinoma and nephrotic syndrom. *N. Eng. J. Med.*, **289,** 520.

Gold, P. (1967) Circulating antibodies against carcinoembryonic antigens of the human digestive system. *Cancer*, **20,** 1663.

Gold, J. M., Freeman, S. O. & Gold, P. (1972) Human anti-CEA antibodies detected by radioimmunoelectrophoresis. *Nature, New Biol.*, **239,** 60.

Hansen, H. J., Lance, K. P. & Krupey, J. (1971) Demonstration of an ion-sensitive antigenic site on carcinoembryonic antigen using zirconyl phosphate gel. *Clin. Res.*, **19,** 143.

HARVEY, S. R., VAN DUSEN, L. R., DOUGLASS, E. D., HOLYOKE, E. C. & CHU, T. M. (1978) Identification of a macromolecule containing an anticarcinoembryonic antigen-reactive substance and immunoglobulin M in human pancreatic cancer. *J. Natl Cancer Inst.*, **61**, 1199.

JOSE, D. G. & SESHADRI, R. (1974) Circulating immune complexes in human neuroblastoma: Direct assay and role in blocking specific cellular immunity. *Int. J. Cancer*, **13**, 824.

KAPSOPOULOU-DOMINOS, K. & ANDERER, F. A. (1979*a*) Circulating carcinoembryonic antigen immune complexes in sera of patients with carcinomata of the gastrointestinal tract. *Clin. Exp. Immunol.*, **35**, 190.

KAPSOPOULOU-DOMINOS, K. & ANDERER, F. A. (1979*b*) An approach to routine estimation of circulating carcinoembryonic antigen immune complexes in patients with carcinomata of the gastrointestinal tract. *Clin. Exp. Immunol.*, **37**, 25.

LOGERFO, P., HERTER, F. P. & BENNETT, F. J. (1972) Absence of circulating antibodies to CEA in patients with gastrointestinal malignancies. *Int. J. Cancer*, **9**, 344.

MCSWEEN, J. M. (1975) The antigenicity of carcinoembryonic antigen in man. *Int. J. Cancer*, **15**, 246.

SJÖGREN, H. O., HELLSTRÖM, I., BANSAL, S. C. & HELLSTRÖM, K. E. (1971) Suggestive evidence that the "blocking antibodies" of tumour bearing individuals may be antigen-antibody complexes. *Proc. Natl Acad. Sci. U.S.A.*, **68**, 1372.

SOROKIN, J. J., KUPCHICK, H. Z. & ZAMCHECK, N. (1973) Carcinoembryonic antigen in colon cancer: Absence in perchloric acid precipitates of plasma. *J. Natl Cancer Inst.*, **51**, 1081.

STEELE, G. J. R., SJÖGREN, H. O. & PRICE, M. R. (1975) Tumor-associated and embryonic antigens in soluble fractions of a chemically-induced rat colon carcinoma. *Int. J. Cancer*, **16**, 33.

TAGLIABUE, A., HERBERMAN, R. B., ARTHUR, L. O. & MCCOY, J. L. (1979) Cellular immunity to tumor-associated antigens of transplantable mammary tumors of C3H/HeN mice. *Cancer Res.*, **39**, 35.

TNM Classification of Malignant Tumors (1978) (Ed. M. Harmer), 3rd Ed. New York: Springer-Verlag.

VON KLEIST, S. & BURTIN, P. (1966) On the specificity of auto-antibodies present in colon cancer patients. *Immunology*, **10**, 507.

VON KLEIST, S., CHAVANEL, G. & BURTIN, P. (1972) Identification of a normal antigen that cross-reacts with the carcinoembryonic antigen. *Proc. Natl Acad. Sci. U.S.A.*, **69**, 2492.

VON KLEIST, S., TROUPEL, S., KING, M. & BURTIN, P. (1977) A clinical comparison between nonspecific cross-reacting antigen and CEA in patients' sera. *Br. J. Cancer*, **35**, 875.

VON KLEIST, S., KING, M. & HAVEMANN, K. (1978) Demonstration of antibodies in patients' sera, directed against nonspecific cross-reacting antigen. *J. Natl Cancer Inst.*, **61**, 1385.

ZÖLLER, M., PRICE, M. R. & BALDWIN, R. W. (1976) Inhibition of cell-mediated cytotoxicity to chemically induced rat tumour and embryo cell extracts. *Int. J. Cancer*, **17**, 129.


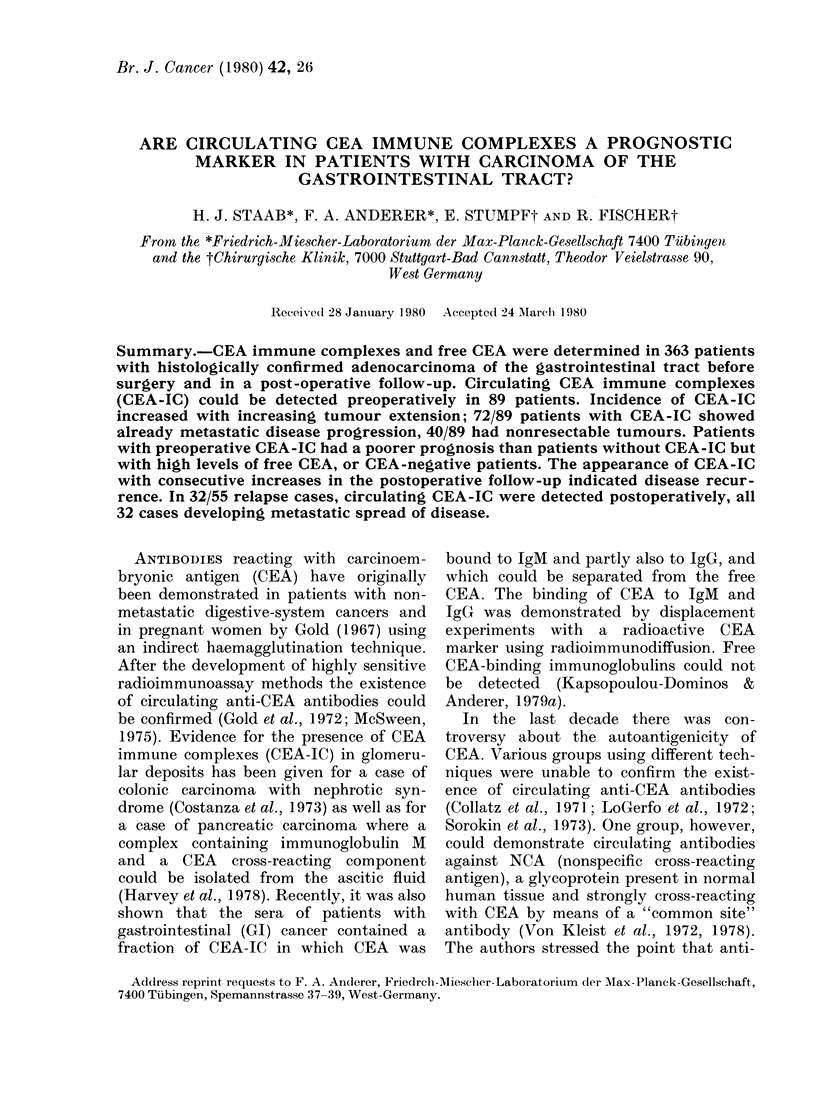

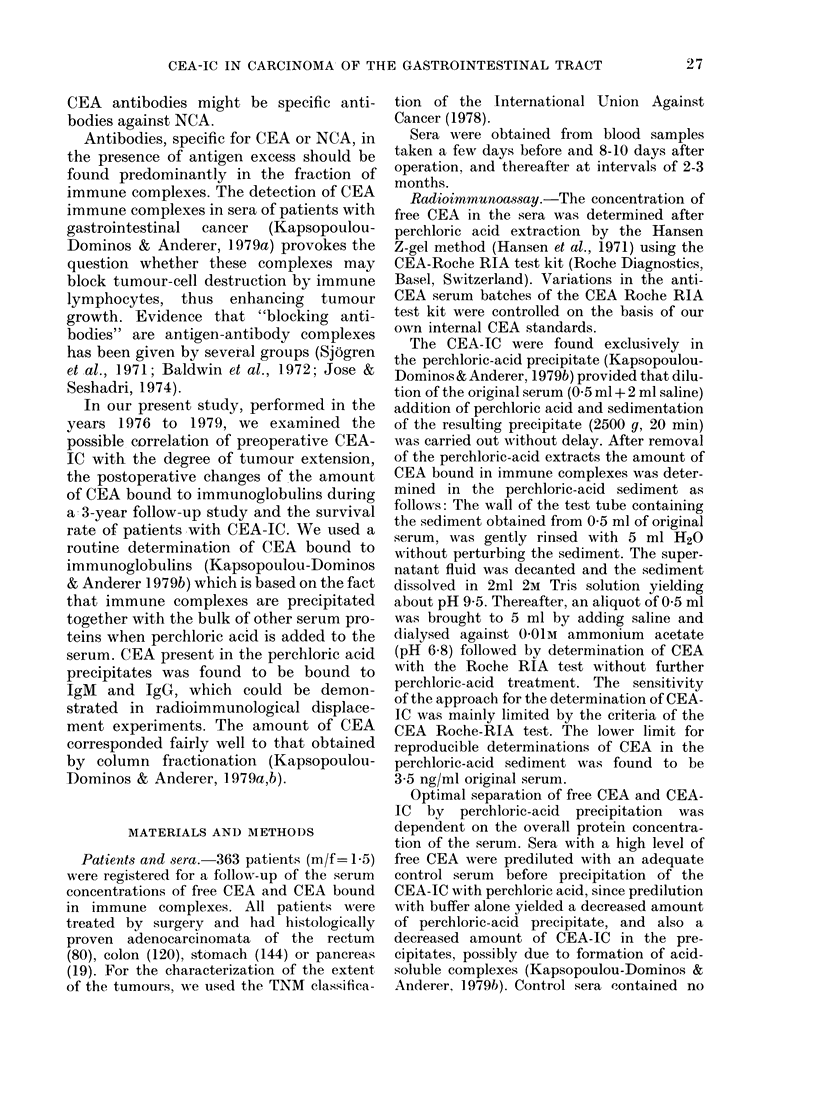

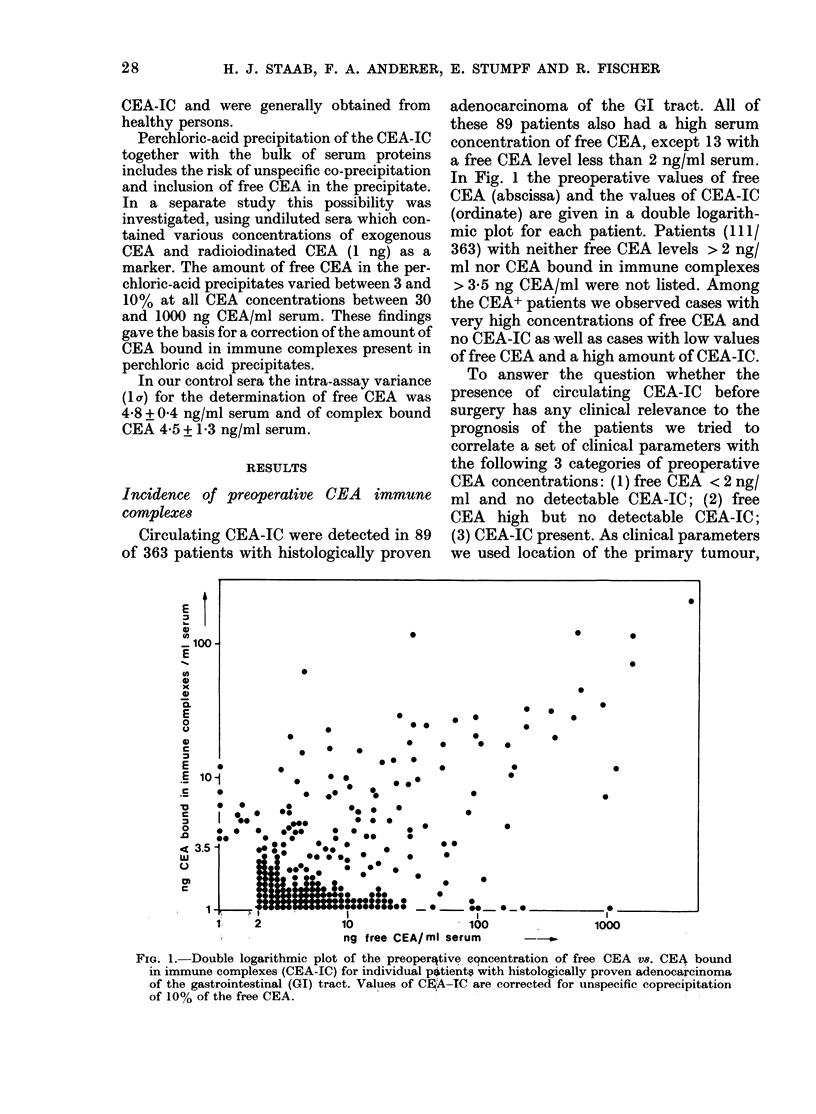

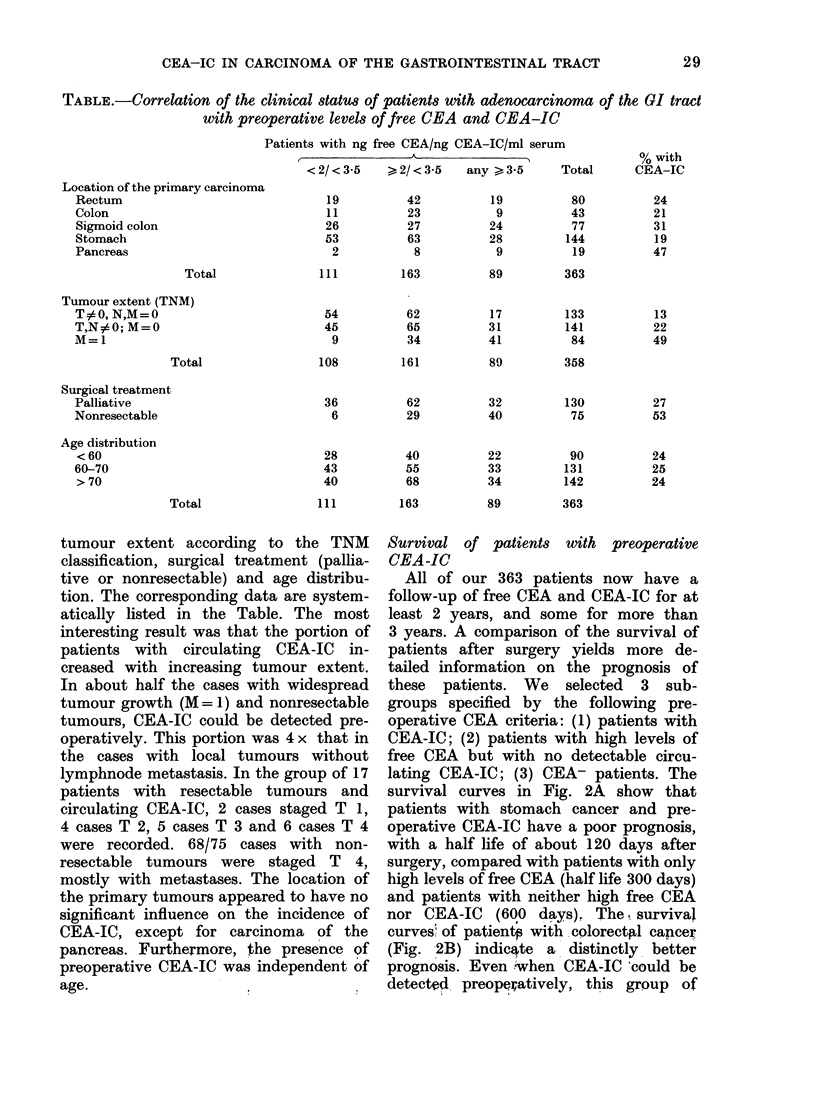

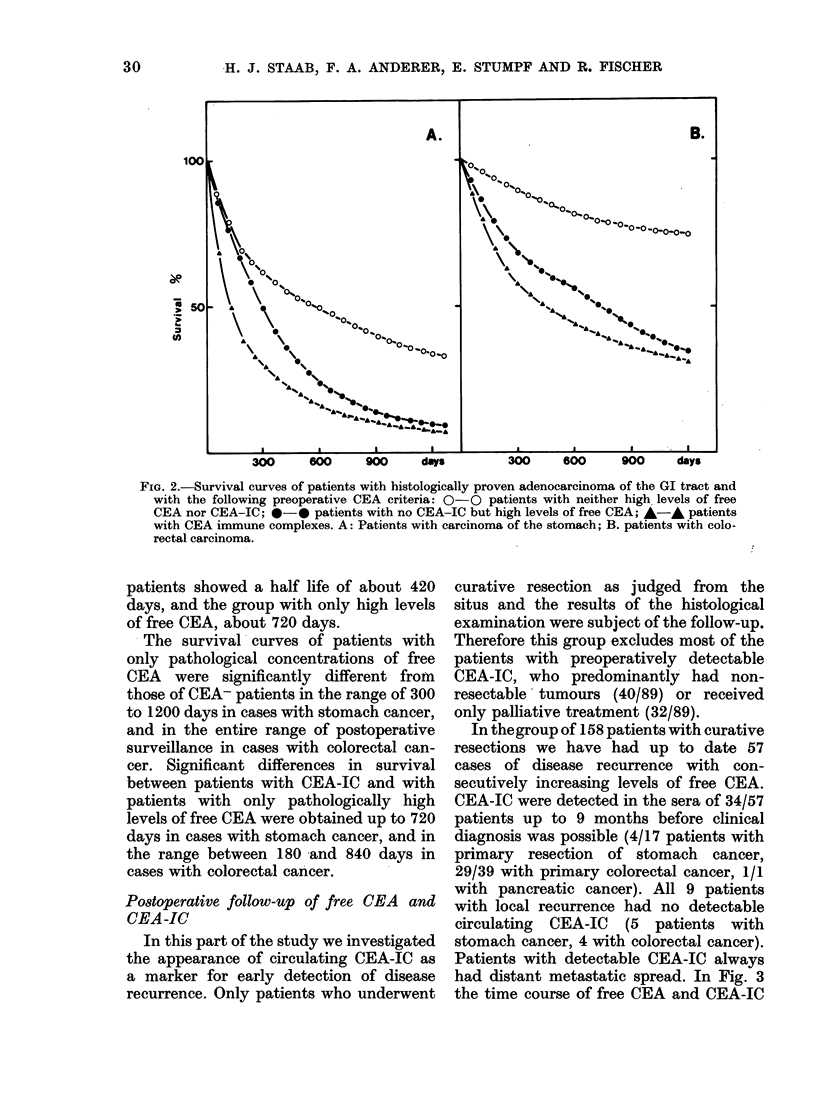

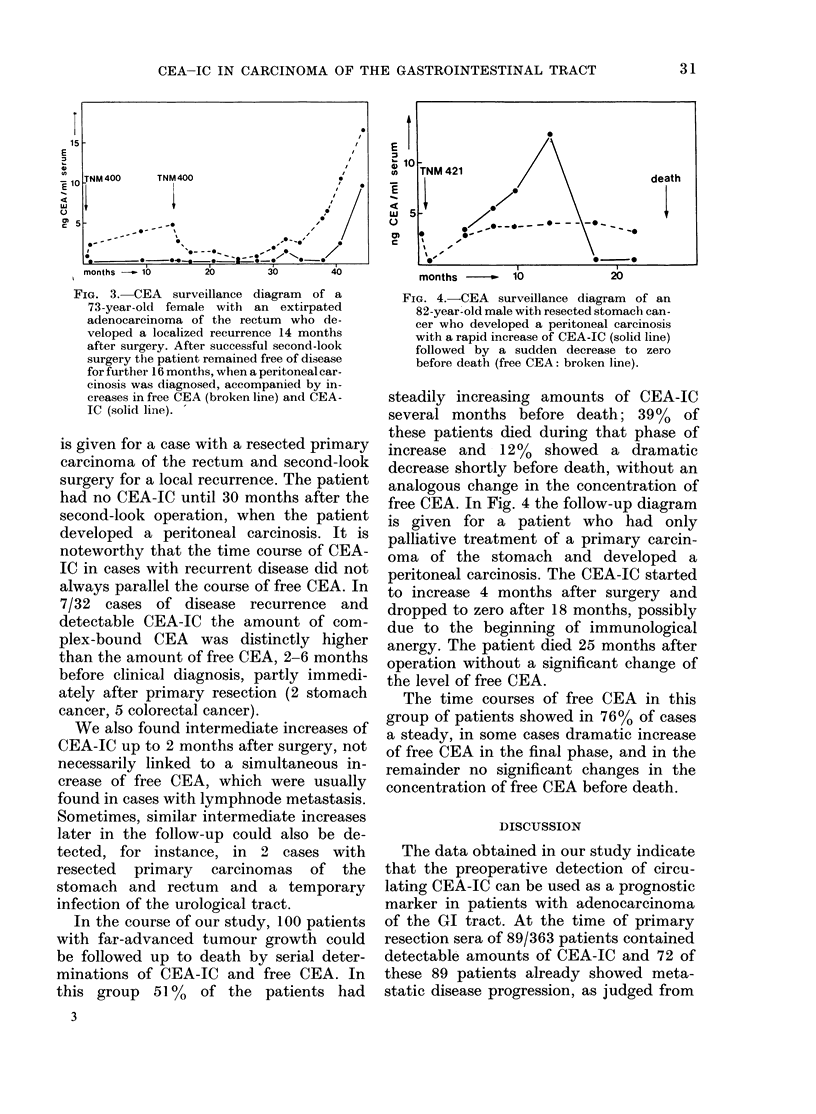

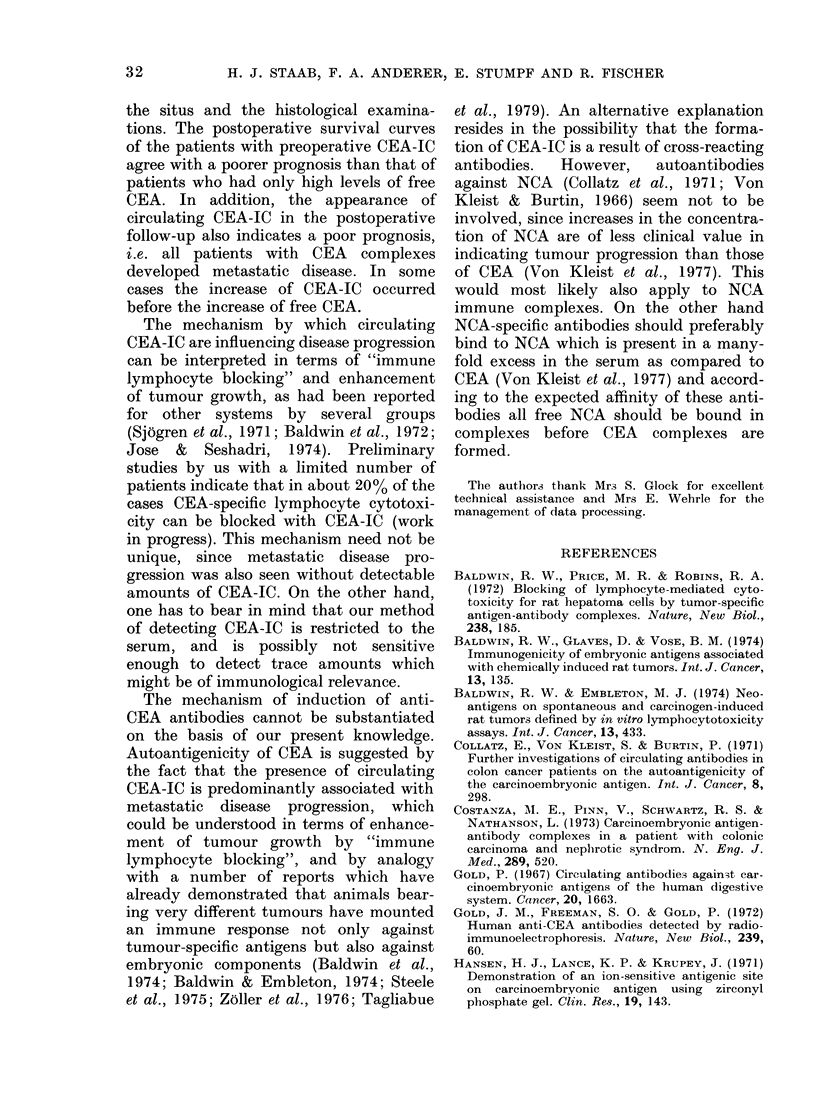

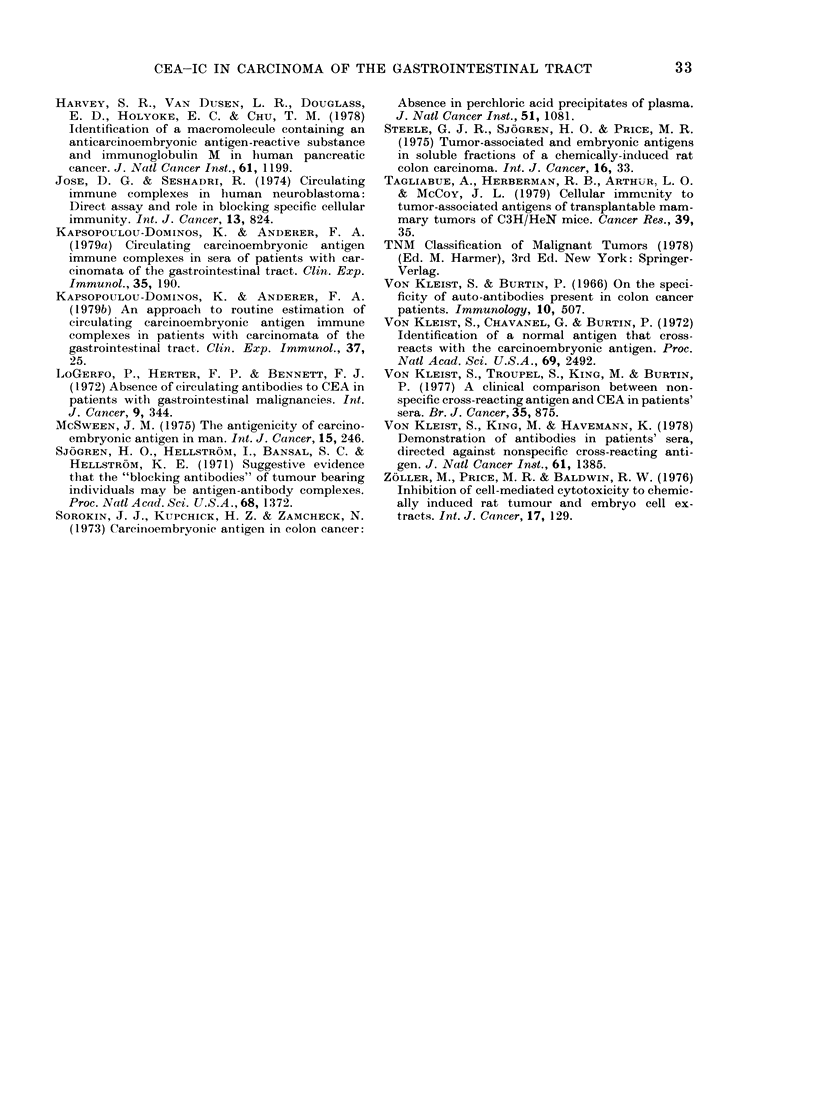


## References

[OCR_00858] Baldwin R. W., Embleton M. J. (1974). Neoantigens on spontaneous and carcinogen-induced rat tumors defined by in vitro lymphocytotoxicity assays.. Int J Cancer.

[OCR_00852] Baldwin R. W., Glaves D., Vose B. M. (1974). Immunogenicity of embryonic antigens associated with chemically induced rat tumours.. Int J Cancer.

[OCR_00845] Baldwin R. W., Price M. R., Robins R. A. (1972). Blocking of lymphocyte-mediated cytotoxicity for rat hepatoma cells by tumour-specific antigen-antibody complexes.. Nat New Biol.

[OCR_00864] Collatz E., Von Kleist S., Burtin P. (1971). Further investigations of circulating antibodies in colon cancer patients: on the autoantigenicity of the carcinoembryonic antigen.. Int J Cancer.

[OCR_00873] Costanza M. E., Pinn V., Schwartz R. S., Nathanson L. (1973). Carcinoembryonic antigen-antibody complexes in a patient with colonic carcinoma and nephrotic syndrome.. N Engl J Med.

[OCR_00883] Gold J. M., Freedman S. O., Gold P. (1972). Human anti-CEA antibodies detected by radioimmunoelectrophoresis.. Nat New Biol.

[OCR_00897] Harvey S. R., Van Dusen L. R., Douglass H. O., Holyoke E. D., Chu T. M. (1978). Identification of a macromolecule containing an anticarcinoembryonic antigen-reactive substance and immunoglobulin M in human pancreatic cancer.. J Natl Cancer Inst.

[OCR_00905] Jose D. G., Seshadri R. (1974). Circulating immune complexes in human neuroblastoma: direct assay and role in blocking specific cellular immunity.. Int J Cancer.

[OCR_00911] Kapsopoulou-Dominos K., Anderer F. A. (1979). Circulating carcinoembryonic antigen immune complexes in sera of patients with carcinomata of the gastrointestinal tract.. Clin Exp Immunol.

[OCR_00926] Lo Gerfo P., Herter F. P., Bennett S. J. (1972). Absence of circulating antibodies to carcinoembryonic antigen in patients with gastrointestinal malignancies.. Int J Cancer.

[OCR_00932] MacSween J. M. (1975). The antigenicity of carcinoembryonic antigen in man.. Int J Cancer.

[OCR_00944] Sorokin J. J., Kupchik H. Z., Zamcheck N. (1973). Brief communication: carcinoembryonic antigen in colon cancer: absence in perchloric acid precipitates of plasma.. J Natl Cancer Inst.

[OCR_00990] Zöller M., Price M. R., Baldwin R. W. (1976). Inhibition of cell-mediated cytotoxicity to chemically induced rat tumours by soluble tumour and embryo cell extracts.. Int J Cancer.

[OCR_00967] von Kleist S., Burtin P. (1966). On the specificity of autoantibodies present in colon cancer patients.. Immunology.

[OCR_00972] von Kleist S., Chavanel G., Burtin P. (1972). Identification of an antigen from normal human tissue that crossreacts with the carcinoembryonic antigen.. Proc Natl Acad Sci U S A.

[OCR_00984] von Kleist S., King M., Havemann K. (1978). Demonstration of antibodies in patients' sera, directed against nonspecific cross-reacting antigen.. J Natl Cancer Inst.

[OCR_00978] von Kleist S., Troupel S., King M., Burtin P. (1977). A clinical comparison between non-specific cross-reacting antigen and CEA in patient's sera.. Br J Cancer.

